# Beware of Selfies: The Impact of Photo Type on Impression Formation Based on Social Networking Profiles

**DOI:** 10.3389/fpsyg.2017.00188

**Published:** 2017-02-16

**Authors:** Nicole C. Krämer, Markus Feurstein, Jan P. Kluck, Yannic Meier, Marius Rother, Stephan Winter

**Affiliations:** Department of Social Psychology: Media and Communication, University of Duisburg-EssenDuisburg, Germany

**Keywords:** selfies, group selfies, sex difference, social networking sites, impression formation, attractiveness, extraversion, narcissism

## Abstract

Users of social networking sites such as Facebook frequently post self-portraits on their profiles. While research has begun to analyze the motivations for posting such pictures, less is known about how selfies are evaluated by recipients. Although producers of selfies typically aim to create a positive impression, selfies may also be regarded as narcissistic and therefore fail to achieve the intended goal. The aim of this study is to examine the potentially ambivalent reception of selfies compared to photos taken by others based on the Brunswik lens model Brunswik (1956). In a between-subjects online experiment (*N* = 297), Facebook profile mockups were shown which differed with regard to picture type (selfie vs. photo taken by others), gender of the profile owner (female vs. male), and number of individuals within a picture (single person vs. group). Results revealed that selfies were indeed evaluated more negatively than photos taken by others. Persons in selfies were rated as less trustworthy, less socially attractive, less open to new experiences, more narcissistic and more extroverted than the same persons in photos taken by others. In addition, gender differences were observed in the perception of pictures. Male profile owners were rated as more narcissistic and less trustworthy than female profile owners, but there was no significant interaction effect of type of picture and gender. Moreover, a mediation analysis of presumed motives for posting selfies revealed that negative evaluations of selfie posting individuals were mainly driven by the perceived motivation of impression management. Findings suggest that selfies are likely to be evaluated less positively than producers of selfies might suppose.

## Introduction

The trend of uploading selfies appears to be a growing form of self-presentation and self-promotion in social networking sites (SNS) such as Facebook. A selfie can be described as a self-portrait that a person has taken of oneself, typically with a smartphone or a webcam, and which is frequently shared with others on social media (Sorokowski et al., 2015). Within the last years, taking selfies has become an extremely popular activity, especially among young people. In a survey, 98% of the 18–24-year-old interviewees stated that they had taken selfies at least once in their lives and 46% revealed that they had shared a selfie that day (Katz and Crocker, 2015). Young adults (aged 20–30) are even more likely to engage in selfie taking and sharing compared to adolescents and adults (Dhir et al., 2016). While researchers have begun to investigate individuals' social and psychological motivations for taking and sharing selfies online (e.g., Weiser, 2015; Sung et al., 2016; Barry et al., 2017; Wang et al., in press), very little is known about the perception of selfies. Despite the apparent popularity of taking selfies, the reception of selfies may seem to be predominantly negative. First empirical evidence for this assumption is presented by Re et al. (2016). As a side result of their empirical investigation on the differences between selfie takers' self-ratings and external raters' judgments, they demonstrated that persons in selfies were rated more negatively than the same persons in photos taken by others. However, as the authors were not focusing on this specific difference, the finding warrants further investigation and needs to be addressed in a systematic study that targets potential mechanisms and explanations for this interesting phenomenon. At first glance, the assumption of negative outcomes may be in contrast to the broad popularity of selfies. For example, Pounders et al. (2016) found that selfie takers are motivated to share selfies in order to create a positive self-image by expressing happiness or a positive physical appearance. More importantly, the assumption of negative outcomes is in contrast with findings that pictures with faces and selfies on Instagram tend to generate a high number of likes (Bakhshi et al., 2014; Souza et al., 2015). However, it could be argued that greater attention and positive feedback from close contacts who wish to maintain a positive relationship with the profile owner (Lee et al., 2014, 2016; Scissors et al., 2016) do not necessarily preclude a negative interpretation by a larger audience.

First results on selfies also suggest that gender is an important variable which might need to be considered when trying to explain the perception and evaluation of selfies. In this respect, several studies indicate a behavioral difference between males and females in taking and sharing selfies, with females being found to be much more active than males (Sorokowski et al., 2015, 2016; Dhir et al., 2016; Sorokowska et al., 2016). These findings on the production of selfies enable hypotheses to be derived regarding the perception of selfies, as observers' evaluations will vary according to their general expectations, which are based on viewing habits.

Another relevant question is whether potential detrimental effects of selfies compared to photos taken by others will also apply to group selfies. Here, mechanisms might differ (a) because less narcissism is attributed when a person is not presenting him/herself alone and (b) because—in general—it has been shown that a person is evaluated as more attractive when she or he is located close to an attractive friend (Kernis and Wheeler, 1981). This has also been demonstrated in a similar form for photos on Facebook (Walther et al., 2008).

The aim of this study is therefore to examine the reception of selfies in comparison to photos taken by other persons while taking sex and number of displayed persons into account. To understand the mechanisms of person perception based on selfies, in line with the general assumptions of the Brunswik lens model (Brunswik, 1956), an array of dependent variables will be assessed that will help to disentangle the attributions made by observers of selfies. With regard to terminology, we will use the term photo for photos that are not taken by any of the shown individuals. If we are not drawing any distinction between selfies and classic photos, we will use the term picture.

### Impression formation

The perceived impressions of individuals' personality in SNSs seem to be largely accurate. For example, Back et al. (2010) found that self-assessment and external assessment ascribed after looking at a social media self-presentation were predominantly consistent. These predictions of personality could be explained by various behaviors in SNSs, which indeed correspond with certain types of personality (e.g., Correa et al., 2010; Moore and McElroy, 2012). The Brunswik lens model (Brunswik, 1956) can be used to describe why and how individuals form impressions based on a limited amount of information when observing others' online behavior. According to this model, it can be reasoned that whenever an individual forms an impression, she or he has several cues or indicators which may or may not apply as aids in the process. In addition, humans tend to use cues systematically even if the cues may possess no real predictive power (Dudycha and Naylor, 1966). The lens model assumes that individuals consider every given piece of information about another person to build an impression about that person's personality. Previous studies investigating impression formation on Facebook based on the lens model discovered that various types of information affect the impression formation process. One study detected a relationship between the number of friends and extraversion insofar as the more friends a person has, the more extraversion is attributed (although only up to the number of ~500 friends), while social attractiveness is rated highest when around 300 friends are displayed, but evaluated lower with fewer or more friends (Tong et al., 2008). Another study similarly showed that the number of friends on Facebook is associated with extraversion, whereas positive affect as well as family-talk in status updates are associated with conscientiousness (Hall and Pennington, 2013). It can be assumed that the type of picture shown is also an important cue that leads to specific attributions about a person's character and personality. In this respect, the specific form of the selfie has already been shown to play a role: Based on different facial expressions or backgrounds, selfies are able to transport personality traits like extraversion, neuroticism and conscientiousness (Qiu et al., 2015). Given that the aim of the present study is not to analyze the differential effects of specific selfie cues but to identify the effects related to this picture form *per se*, the next section refers to different types of pictures more generally.

### Type of picture

To explain potential differences in ratings between selfies compared to photos taken by others, the warranting principle (Walther and Parks, 2002) can be drawn upon. Simply put, this principle assumes that individuals mistrust information that can easily be manipulated. In the context of selfies, one can argue based on the warranting principle that individuals should distrust selfies to a greater extent than photos because selfies are apparently easier to manipulate than photos. Research investigating the warranting principle in the context of Facebook found that information generated by individuals other than the profile owner can increase the profile owner's social and task attractiveness and credibility (Walther et al., 2008) and his or her physical attractiveness as well as extraversion and introversion, respectively (Walther et al., 2009).

Besides the warranting principle and in relation to the lens model, it can be assumed that recipients will be able to attribute those personality characteristics that are actually related to posting selfies. For instance, Sorokowska et al. (2016) identified extraversion to be a predictor of selfie-posting behavior. The authors concluded that extroverted individuals might use selfies as a means to keep friends informed about oneself. In addition, Kim and Chock (in press) identified extraversion to be a predictor of posting group selfies, whereas narcissism predicted greater levels of posting solo selfies. Additionally, extroverts are often found to be more active on SNSs than introverts (Nadkarni and Hofmann, 2012), which might also explain differences in posting behavior. Another personality trait that might be associated with sharing selfies in SNSs is openness to new experiences. Researchers have found that individuals who score high on openness participate in many different activities in SNSs such as Facebook (Ross et al., 2009; Gosling et al., 2011), which could include uploading selfies. Moreover, posting photos might be seen as “outdated,” whereas posting selfies is clearly a newer phenomenon and might therefore lead to attributions of higher openness. However, Moore and McElroy (2012) did not find any significant results suggesting that openness leads to a specific kind of behavior on Facebook.

Based on the findings regarding personality traits and SNS behavior (Ross et al., 2009; Gosling et al., 2011; Nadkarni and Hofmann, 2012; Sorokowska et al., 2016), we assume that recipients ascribe those characteristics which are actually related to selfie-posting, and hypothesize that individuals in selfies are rated (*H1a*) as more extroverted and (*H1b*) as more open than individuals in photos taken by others. Furthermore, based on the warranting principle (Walther and Parks, 2002) and the aforementioned study findings (Walther et al., 2008, 2009; Re et al., 2016), we assume that persons in selfies are rated as (*H1c*) less physically attractive, (*H1d*) less socially attractive, (*H1e*) more narcissistic, and (*H1f*) less trustworthy than persons in photos taken by other persons.

### Effects of gender of the profile owner

Previous studies have shown that there might be a gender difference in SNS usage, especially regarding self-presentation (Haferkamp et al., 2012). It has been found that male users tend to use SNS primarily for information purposes, whereas female users place greater value on a diverse self-presentation. These findings are supported by studies indicating that female Facebook users are more active and put greater effort into impression management via their profile pictures (McAndrew and Jeong, 2012). Moreover, females seem to put more effort into emotional expressions in their posted pictures than males (Zheng et al., 2016). Studies investigating selfie-posting behavior suggest a gender-related distortion in selfie-sharing behavior, with females posting twice as many selfies as males (Sorokowski et al., 2015, 2016; Sorokowska et al., 2016). In addition, Dhir et al. (2016) found that female individuals not only post more selfies in SNSs but also take more selfies in general than male individuals. In sum, these observations lead to the assumption that sharing selfies in SNSs is a behavior that can be expected more from females than from males. This difference in behavior alone might lead to different evaluations of the same behavior according to expectations. For example, research on the perception of smiles shows that smiling behavior, when it is not expected (i.e., from male individuals, who do not usually smile as much as female individuals), leads to more positive evaluations than when it is the perceived norm (Deutsch et al., 1987). Moreover, past studies have shown that female and male selfie-takers differ concerning some personality attributes. Results on the production and sharing of selfies revealed that narcissism (Fox and Rooney, 2015; Sorokowski et al., 2015; Weiser, 2015), psychopathy (Fox and Rooney, 2015), and histrionic personality (Sorokowski et al., 2016) were able to predict the number of posted selfies, particularly among men.

If taking and sharing selfies can be seen as a more expected behavior of female individuals than male individuals, and if, according to the lens model, participants are able to infer the personality attributes related to selfie posting (Fox and Rooney, 2015; Sorokowski et al., 2015; Weiser, 2015), participants should rate males in selfies (*H2a*) higher on narcissism and (*H2b*) as less trustworthy than females in selfies.

### Effects of single persons vs. groups in pictures

So far, we have focused on the perception of selfies that one individual has taken of her- or himself. In addition to single selfies, it has been indicated that group selfies, i.e., selfies that present at least two persons, are the most popular picture category to share on Instagram (Hu et al., 2014). Moreover, Wang et al. (in press) showed that participants prefer Facebook for sharing group selfies rather than other social media platforms (e.g., Instagram, Twitter, Snapchat). To our knowledge, no previous study has examined the distinct perception and evaluation of group selfies and single selfies.

As it is suggested that in the context of online presence, persons are evaluated by any given social information (Walther, 2007), one could expect the perception of group selfies to differ from single selfies insofar as a group selfie includes more social cues (e.g., relationship with other people, behavior in groups). One important social characteristic that is often used to gain a first impression about a person is physical attractiveness, and the assessment of facial attractiveness is highly influenced by the observation of other faces in the environment (Pegors et al., 2015). In this context, it was shown that a person is regarded as more attractive when she or he is located close to an attractive friend (Kernis and Wheeler, 1981). Walther et al. (2008) found this effect to be also true for photos on Facebook—although it should be noted that in this study, the faces of the others were not as close as in a joint or combined photo. Likewise, Walker and Vul (2014) found evidence that faces appear to be more physically attractive when persons are photographed in a group rather than pictured alone. To explain their findings, the authors referred to research on ensemble coding in the visual system as well as the characteristics of average faces (e.g., Ariely, 2001; Langlois and Roggman, 1990; Brady and Alvarez, 2011) and argued that an interplay of three cognitive phenomena is causative: (a) various objects are calculated as an ensemble by the visual system, (b) the average of this ensemble biases individual objects, and (c) average faces are evaluated as more physically attractive.

In the sense of the halo effect, an attractive physical appearance has been shown to have numerous positive side effects. Physical attractiveness is linked to the attribution of positive character traits such as intelligence, healthiness, popularity and social skills (for a review see Langlois et al., 2000). This phenomenon is also called the attractiveness halo effect (Kaplan, 1978). Besides this effect, there might also be a more direct route of attributing social attractiveness on Facebook: Hong et al. (2012) found evidence that a higher number of social cues in Facebook profile pictures is positively related to social attractiveness and popularity.

Based on the findings that people in groups are considered as more attractive, and are also perceived as more socially attractive due to the attractiveness halo effect, we hypothesize further that (*H3a*) individuals in group photos are perceived as more physically attractive, and (*H3b*) individuals in group photos are perceived as more socially attractive than individuals in single photos. In line with this, we also assume that (*H3c*) individuals in group selfies are perceived as more physically attractive and (*H3d*) individuals in group selfies are perceived as more socially attractive than individuals in single selfies.

### Influence of perceived motives

Despite a growing scientific interest in the mechanisms underlying selfie-taking and -sharing behavior, the motivational aspect of such behavior has largely been neglected. While motives for using SNS have been identified based on the uses and gratification approach (e.g., maintaining existing relationships, entertainment, impression management, need to belong; Joinson, 2008; Krämer and Winter, 2008; Sheldon, 2008; Papacharissi and Mendelson, 2011; Smock et al., 2011; Nadkarni and Hofmann, 2012; Tosun, 2012), Sung et al. (2016) began to explore the motivational factors behind selfie-posting behavior. Based on uses-and-gratification assumptions, they emphasized the importance of motivation as a determinant of SNS usage and also as necessary for a better understanding of the mechanisms behind selfie-posting behavior. Four unique motivations were identified by Sung et al. (2016): Attention seeking, communication, archiving, and entertainment. Regarding our research goal of determining the factors which affect the perception of selfies, however, it is more important to not only identify selfie takers' and sharers' actual motivation, but to also assess the motives that are attributed by the recipients. Previous research on person perception based on self-disclosure on SNS demonstrates that the evaluation of an (intimate) posting and its sender is dependent on the reasons for posting that are attributed by the recipients. If impression management is assumed to be the motivation for posting an intimate message, this is more detrimental than when the ascribed reason is support seeking (Krämer et al., 2014). On a theoretical level, this can be explained by the Brunswik lens model (Brunswik, 1956): When forming an impression of an individual's personality, it is very likely that perceivers take motives into account in order to generate a more accurate perception of the person. Accordingly, we suggest that the attributed motives mediate the relationship between the assessment of personality and the type of picture.

Therefore, in a first step, we argue that it is necessary to systematically assess which motives observers assume when perceiving selfies. In order to fill this gap in the literature, we ask (*RQ1*) which motivations for picture sharing are attributed to the picture producers.

With regard to the assessment of personality, it seems most reasonable to focus on narcissism, as narcissism has been found to be relevant with regard to both the production and the perception of selfies. Concerning perception, narcissism has been shown to be strongly related to selfie-related activities (Fox and Rooney, 2015; Sorokowski et al., 2015; Weiser, 2015), to picture-related activities (Kapidzic, 2013), and to Facebook usage in general (Mehdizadeh, 2010). In terms of the perception of selfies, Re et al. (2016) suggest that perceived narcissism might be causative for their side finding that people in selfies are perceived as less positive as people in photos. This, however, has to be analyzed more systematically and taking the presumed motives for the selfie-posting behavior into account.

Therefore, we hypothesize that (*H4*) the relationship between type of picture and ascribed narcissism (as stated in *H1e*) is mediated by the presumed motives for picture posting.

## Methods

### Design

In this study, a 2 (type of picture: Selfie vs. photo taken by other persons) x 2 (gender of pictured person: Female vs. male) x 2 (number of pictured persons: Single vs. group) between-subjects design was used and tested in an online survey (*N* = 297).

### Stimulus material

**Figure 1:**
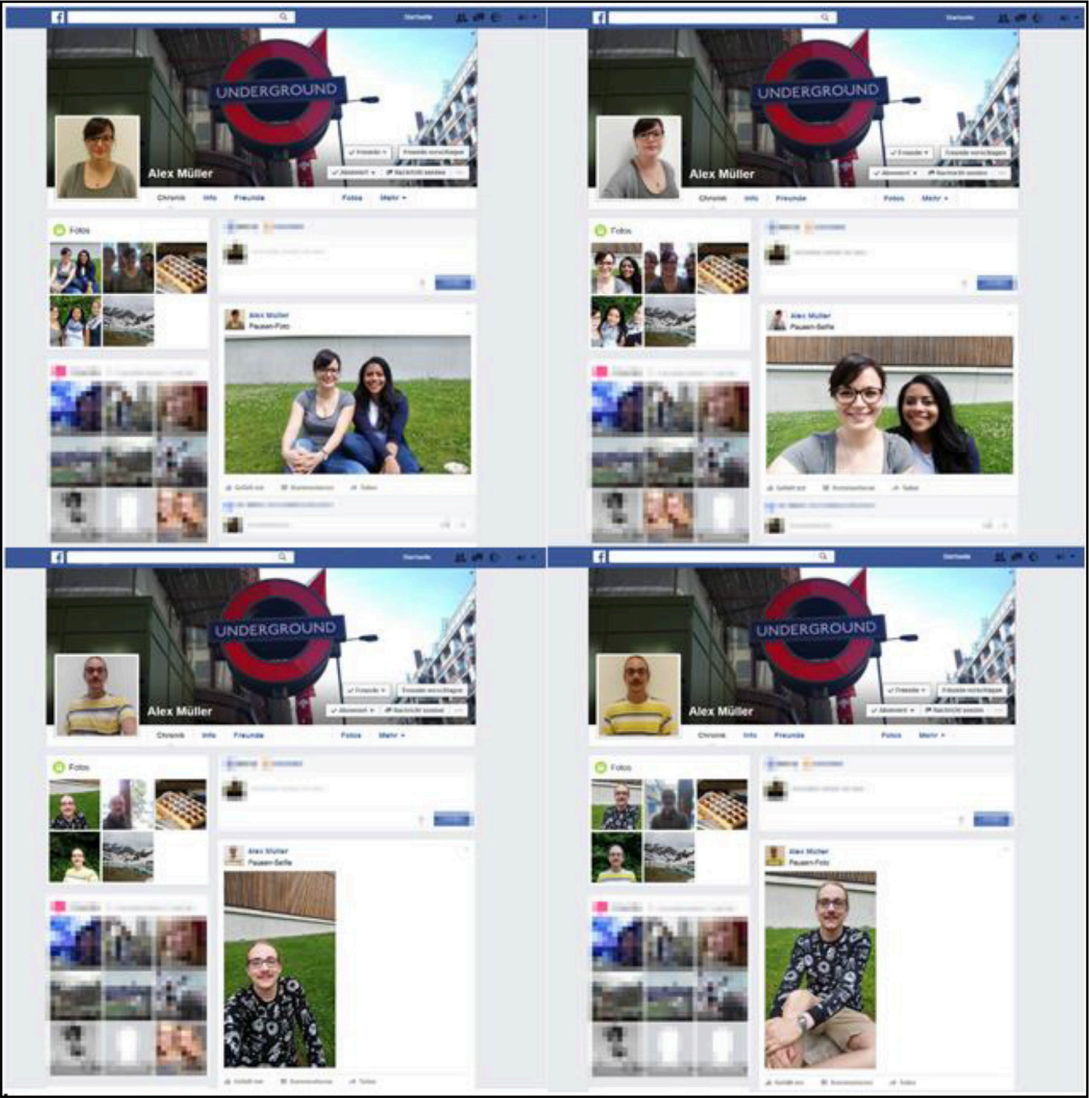
**Partial Facebook Mockups for conditions:** Female Group Photo **(upper left)**, Female Group Selfie **(upper right)**, Male Single Selfie **(bottom left)**, and Male Single Photo **(bottom right)**.

The online survey consisted of a Facebook profile mockup that was presented at the beginning and followed by questionnaires. In order to ensure that results would not only hold for one specific target person, six actors (three female actors, three male actors) were presented identically in various pictures, including the profile picture and three posted pictures, for each condition. Selfies were taken by the actors themselves and photos were taken by the experimenters. A smartphone (Samsung Galaxy S5) was used to take these pictures in order to remain authentic. Three different locations were chosen to take the pictures. At every location, a single photo and a single selfie were taken for each person. Furthermore, group pictures and group selfies were taken showing all three actors at two of the venues and two of the actors at the third venue. Facial expressions were moderately friendly for all conditions. Clothing was altered at each location to create an impression of authenticity. To make the pictures and selfies comparable, the posing for every condition was the same and was instructed by the experimenters (e.g., no specific selfie-posing such as duckfacing). Besides the profile pictures and the three person-related pictures that were manipulated, the Facebook profile mockup contained three additional neutral photos showing a dessert (waffles) and a mountain landscape as wall postings as well as a typical underground sign from London as the cover photo in order to create a realistic setting. For the neutral photos, we referred to Hu et al. (2014), who revealed the most commonly posted photo contents on Instagram. We chose those common contents which we considered as gender neutral. In summary, the Facebook profile mockups each consisted of 1 neutral cover photo, 1 personal profile picture, 3 person-related pictures, and 2 neutral pictures as wall postings. In the first person-related picture, the actors for all conditions were sitting in a green area with a gray building in the background on campus. In the second person-related picture, the actors were standing in front of a forest and green bushes, and in the third person-related picture, they were sitting on a window sill. The profile picture was always taken in front of a white background. The profile owner was named “Alex Müller” in all profile mockups because “Alex” is a German unisex first name and “Müller” is a very common German surname. Irrelevant information, like comments, likes or time of posting, was implemented, but was blurred to avoid unwanted effects. For an illustration of the material, see Figure 1. In total, 24 different mockups were set up (8 conditions with three different actors each), to which participants were randomly allocated.

### Sample

436 participants began the study, of whom 127 were excluded due to incomplete data. A further eight datasets were not considered further because the participants reported that they knew the person on the Facebook profile. Another four datasets were excluded because the Facebook profile was observed for less than 5 s.

The final sample thus consisted of a total of 297 participants (205 females, 91 males, 1 did not specify gender) aged 15 to 66 (*M* = 27.34, *SD* = 7.98, 1 missing value). The sample was split into three different age groups: The first age cohort (*n* = 231) was aged between 15 and 29 years (*M* = 23.95, *SD* = 2.81, 77.78% of the sample), and reported taking the highest number of selfies per week (*M* = 1.72, *SD* = 4.42). The second age cohort (*n* = 54) was aged between 30 and 49 years (*M* = 35.85, *SD* = 5.20, 18.18% of the sample) and stated taking fewer selfies per week (*M* = 0.98, *SD* = 2.62) than the first cohort. The third age cohort (*n* = 12) was aged between 50 and 66 years (*M* = 54.42, *SD* = 4.70, 4.04% of the sample). These individuals took the lowest amount of selfies (*M* = 0.25, *SD* = 0.45). Each of the 24 mockups was rated at least 12 or 13 times (resulting in every condition having at least been viewed by 36 participants). Most interviewees (88.2%) stated that they had a Facebook account. Participants were primarily recruited online via several forums and Facebook groups, but were also addressed personally at a large German university. As a supplementary incentive to participate in the study, they were able to take part in a raffle to win gift vouchers.

### Measures

Each participant completed all of the following questionnaires, which were adapted to the particular conditions (e.g., “he”/“she”; “selfie”/“photo”). All the English-language questionnaires were translated into German.

#### Trustworthiness

To assess the participants' perception of the profile owner's credibility, the five-item trustworthiness subscale of the Source Credibility Scale (SCS; Ohanian, 1990) was used. The SCS consists of bipolar items rated on a seven-point Likert scale (e.g., “trustworthy”—“untrustworthy”). The internal consistency (Cronbach's alpha) was 0.89.

#### Attractiveness

The Interpersonal Attraction Scale (McCroskey and Richmond, 1979) measures different types of attraction on a seven-point Likert scale (from 1 = *strongly disagree* to 7 = *strongly agree*). In the present study, the social attraction and the physical attraction subscales were used. The social attraction subscale consists of 12 items (e.g., “likeable”—“unlikeable”) and its internal consistency (Cronbach's alpha) was 0.85 for both adapted versions for females and males. The physical attraction subscale comprises 11 items (e.g., “attractive”—“unattractive”) and its internal consistency was 0.94, for both versions for females and males.

#### Narcissism

To assess the participants' impression of the profile owner's level of narcissism, a German version of the short Narcissistic Personality Inventory (NPI-15; Spangenberg et al., 2013) was slightly adapted. The original NPI-15 measures an individual's perceived narcissism of the self, which is why an adjustment to the impression of the profile owner was necessary. One item had to be excluded because it would have been pointless to rate an unfamiliar person with this specific item. The NPI-15 consists of bipolar items, one of which measures perceived narcissism and its internal consistency (Cronbach's alpha) was 0.84. To ensure that the NPI-15 is able to measure another person's perceived narcissism, a single item called “the profile owner is narcissistic” was also presented on a seven-point Likert scale (from 1 = *does not apply at all* to 7 = *does definitely apply*). The correlation (*r* = 0.46, *p* < 0.001) between the NPI-15 sum score and this single item provides a hint that it can be used to assess an individual's impression of another person's narcissism.

#### Openness and extraversion

The participants' perceptions of the profile owner's extent of openness and extraversion were measured by the openness and extraversion subscales of the German version of the Big Five Inventory (BFI-44; Lang et al., 2001). Items of the BFI-44 are rated on a five-point Likert scale (from 1 = *does not apply at all* to 5 = *does definitely apply*) and were adapted to the perceived personality of the profile owner. The internal consistency (Cronbach's alpha) was 0.79 and 0.82 for openness and extraversion, respectively.

#### Presumed motives

To find out which motivational factors the participants attributed to the profile owners' photo- and selfie-sharing behavior, a questionnaire with 20 items (six-point Likert scale from 1 = *strongly disagree* to 6 = *strongly agree*) was used, and was the same for the selfie condition and the photo condition (Szczuka et al., 2015). According to the condition to which the participants were assigned, the words “selfie” and “photo” were interchanged. Presented motives included a variety of possible attributions (e.g., “I think the profile owner shares selfies to present his body” or “I think the profile owner shares photos because she feels lonely”).

On the basis of the gained data (*N* = 297), we performed two factor analyses in order to reduce the quantity of items and to extract the number of factors. A priori, three items were excluded after examining the descriptive values concerning item difficulty. An exploratory factor analysis (EFA) with principal component analysis and varimax rotation was then conducted. Horn's parallel analysis (Horn, 1965) was used for selecting the appropriate number of factors to retain. The results suggested a three-factor solution. In the next step, EFAs with principal axis analysis and promax rotation were computed in order to consider factor loadings. Items with low loadings on the main factor (<0.50) and/or high loadings on the other factors (>0.20) were removed progressively to improve the quality of the questionnaire. Moreover, two items were excluded due to contextual considerations. This procedure resulted in a three-factor solution with 11 remaining items and good reliability (Cronbach's α = 0.87). In line with our assumption, the first factor (5 items, Cronbach's α = 0.84) revealed items that are mostly related to impression control and was named “impression management.” As the second factor (3 items, Cronbach's α = 0.86) consists of items related to feelings of loneliness and insecurity, it was named “negative emotions.” The third factor (3 items, Cronbach's α = 0.79) was called “coquetry,” because the items deal with the presentation of physical appearance. A significant correlation was found between all factors (“impression management” and “negative emotions: *r* = 0.40, *p* < 0.001, “impression management” and “coquetry”: *r* = 0.46, *p* < 0.001 and “negative emotions” and “coquetry”: *r* = 0.44, *p* < 0.001). Items, factor loadings and descriptive values are presented in Table 1.

**Table 1:** **Descriptive values of items and Factor loadings for EFA with principal axis analysis**.

**Item**	**Factor**			***M***	**(*SD*)**
	**1**	**2**	**3**		
**IMPRESSION MANAGEMENT**					
To get attention	**0.863**	−0.006	−0.029	3.85	(1.31)
To be represented positively	**0.787**	−0.041	−0.049	4.23	(1.30)
To control other people's impressions about her-/himself	**0.673**	0.032	0.107	3.53	(1.43)
To be liked	**0.631**	0.036	−0.016	4.08	(1.29)
To receive feedback	**0.586**	−0.005	0.066	3.74	(1.38)
**NEGATIVE EMOTIONS**					
When she/he has self-doubts	0.019	**0.900**	0.006	2.25	(1.20)
When she/he feels lonely	0.024	**0.860**	−0.066	2.55	(1.38)
When she/he feels insecure	−0.045	**0.702**	0.094	2.23	(1.20)
**COQUETRY**					
To present her/his haircut	−0.073	0.014	**0.914**	2.27	(1.30)
Because she/he is vain	0.057	−0.029	**0.821**	2.27	(1.32)
When she/he changes her/his look	0.076	0.043	**0.509**	2.86	(1.47)

*All items started with “I think the profile owner shares photos/selfies of her-/himself …”. Values of the main factors are in bold*.

#### Additional measurements

To find out what people think about the profile owners in general, a semantic differential was used, consisting of 14 items with a seven-point Likert scale. Items included, for example “helpful—uncooperative” or “dominant—inferior.” To achieve proper factors, we performed the same procedure as described for the presumed motives. The EFA, additionally taking into account Horn's parallel analysis (Horn, 1965), suggested a two-factor solution. After excluding items based on low loadings on the main factor and high loadings on the other factor, 11 items remained on two factors, with a good reliability (Cronbach's α = 0.80). The first factor (7 items, Cronbach's α = 0.90) was named “self-seeking” and was closely related to characteristics of narcissism. The other factor (3 items, Cronbach's α = 0.59) can be summarized by “authority.” Due to the poor reliability of the factor authority, this factor was excluded from further analyses. As the factor self-seeking seemed to be appropriate as a supplementary measure of narcissism, it was used as an additional dependent variable. Items, factor loadings and descriptive values are presented in Table 2. Moreover, participants answered a few general questions concerning selfies and provided demographic data.

**Table 2:** **Descriptive values of items and Factor loadings for EFA with principal axis analysis (*N* = 297)**.

**Item**	**Factor**		***M***	**(*SD*)**
	**1**	**2**		
**SELF-SEEKING**				
Uncooperative	**0.815**	0.082	2.90	(1.05)
Haughty	**0.788**	0.081	2.84	(1.28)
Intransigent	**0.786**	0.132	3.03	(1.04)
Arrogant	**0.764**	−0.215	3.36	(1.19)
Disrespectful	**0.740**	0.146	2.99	(1.06)
Egoistic	**0.720**	0.011	3.58	(0.99)
Egocentric	**0.680**	−0.254	3.69	(1.35)
**AUTHORITY**				
Weak	0.156	**0.718**	3.76	(1.15)
Inferior	−0.265	**0.588**	4.31	(1.01)
Passive	0.078	**0.490**	3.08	(1.37)

*Values of the main factors are in bold*.

The general questions addressed on the one hand the general attitude toward selfies and photos using six items rated on a five-point Likert scale (from 1 = *strongly disagree* to 5 = *strongly agree*), and on the other hand how many selfies and photos participants take and post weekly. Example items on general attitude toward selfies and photos were: “I would never post a selfie of mine on Facebook” or “taking selfies is really fun.” Items for taking and posting selfies and photos were “How many selfies do you take weekly?” and “How many photos of yourself do you post weekly?” As a manipulation check, the participants were also asked how many selfies and how many “photos of persons (not selfies)” they had seen on the presented Facebook profile. With these questions, we aimed to ensure that the stimulus material served its purpose and participants recognized the pictures, according to the conditions, as photos taken by another person or as selfies. Moreover, participants were asked whether they knew any of the presented persons. If participants stated that they knew one of the shown actors, they were excluded from the analysis, as we expected that this would have a significant effect on the evaluation of the pictured person.

### Procedure

The online survey was implemented using SoSci Survey (Leiner, 2014) and was provided via www.soscisurvey.de. The procedure took ~10 min. After a general introduction, the participants were told that they were going to see a Facebook profile of a person. They were free to determine for how long they would look at the Facebook profile but were instructed to build an impression of the person. The questionnaires began with the assessment of attractiveness followed by the evaluation of credibility, the general personality descriptions, narcissism, openness, and extraversion. Subsequently, the participants were asked to rate potential motives for the selfie-/photo-sharing behavior of the profile owner. At the end of the experiment, the participants were questioned about their general attitude toward selfies as well as their own selfie-taking behavior. Additionally, the manipulation check was administered. In a debriefing, the participants were informed about the purpose of this study and had the chance to enter their email address in order to take part in the prize draw.

## Results

All analyses were conducted using IBM SPSS 22.0. Before testing the hypotheses, the descriptive values of the additional measurements were computed. The means for average weekly taken selfies by the participants (*M* = 1.53, *SD* = 4.07) were higher than for posting selfies in the same period (*M* = 0.19, *SD* = 1.26). In line with this, the means for average weekly taken photos of oneself (*M* = 3.88, *SD* = 9.77) were higher than for posted photos of oneself (*M* = 0.34, *SD* = 1.81). The manipulation check revealed that in the photo condition (*n* = 148), the participants believed on average that they had seen 3.49 photos (*SD* = 1.41) and 2.35 selfies (*SD* = 1.38). In the selfie condition (*n* = 149), the participants thought on average that they had seen 3.86 selfies (*SD* = 1.05) and 2.44 photos (*SD* = 1.44). These results are worthy of discussion, as each participant was only presented with either selfies or photos, but not both in one profile. However, focusing on the differences between the two conditions, a *t*-test for unrelated samples revealed that participants remembered significantly more selfies in the selfie condition [*t*_(295)_ = 10.59, *p* < 0.001] than in the photo condition and likewise more photos in the photo condition than in the selfie condition [*t*_(295)_ = −6.34, *p* < 0.001]. These results will be highlighted in the discussion.

### Differences between perceptions

To analyze the hypotheses *H1a*–*H3b*, a MANOVA was performed with the between-subject variables type of picture, gender, and number of pictured persons. The means, standard deviations and confidence intervals of the dependent variables perceived narcissism, perceived trustworthiness, perceived openness, perceived extraversion, as well as perceived social and physical attractiveness can be seen in Table 3.

**Table 3:** **MANOVA: Proportion of the dependent variables for the between-subject conditions (*N* = 297)**.

**Characteristic**	**Type of picture**		**Number of people shown**		**Gender**	
	**Photo (*n* = 148)** ***M* (*SD*),** **[95% CI]**	**Selfie (*n* = 149)** ***M* (*SD*),** **[95% CI]**	**Single (*n* = 148)** ***M* (*SD*),** **[95% CI]**	**Group (*n* = 149)** ***M* (*SD*),** **[95% CI]**	**Female (*n* = 149)** ***M* (*SD*),** **[95% CI]**	**Male (*n* = 148)** ***M* (*SD*),** **[95% CI]**
Perceived narcissism	16.59 (2.65), [16.08, 17.11]	18.50 (3.62), [17.99, 19.01]	17.49 (3.32), [16.99, 18.01]	17.61 (3.31), [17.09, 18.10]	17.16 (3.08), [16.65, 17.67]	17.94 (3.49), [17.43, 18.45]
Perceived trustworthiness	26.48 (4.25), [25.74, 27.20]	24.13 (4.97), [23.41, 24.87]	24.88 (4.76), [24.13, 25.60]	25.72 (4.74), [25.01, 26.47]	26.01 (5.15), [25.30, 26.76]	24.58 (4.23), [23.84, 25.31]
Perceived openness	27.09 (4.56), [26.35, 27.81]	25.54 (4.48), [24.81, 26.27]	26.29 (4.76), [25.55, 27.01]	26.33 (4.40), [25.62, 27.07]	26.89 (4.54), [26.17, 27.62]	25.72 (4.55), [25.00, 26.45]
Perceived extraversion	21.94 (4.15), [21.21, 22.65]	24.00 (4.68), [23.27, 24.71]	23.01 (4.54), [22.28, 23.72]	22.93 (4.54), [22.20, 23.64]	23.13 (4.40), [22.39, 23.83]	22.82 (4.67), [22.09, 23.53]
Perceived physical attractiveness	51.25 (13.48), [49.18, 53.27]	49.83 (13.77), [47.77, 51.85]	50.28 (13.74), [48.08, 52.17]	50.80 (13.54), [48.87, 52.94]	55.76 (11.55), [53.73, 57.80]	45.28 (13.56), [43.22, 47.31]
Perceived social attractiveness	62.70 (9.85), [61.03, 64.32]	59.42 (10.84), [57.78, 61.06]	60.38 (9.66), [58.67, 61.96]	61.73 (11.21), [60.14, 63.42]	63.14 (10.20), [61.52, 64.80]	58.96 (10.35), [57.29, 60.58]
Perceived self-seeking	20.51 (5.86), [19.55, 21.48]	24.24 (6.20), [23.27, 25.20]	22.86 (6.47), [21.91, 23.84]	21.91 (6.13), [20.91, 22.84]	21.58 (6.60), [20.59, 22.52]	23.19 (5.91), [22.23, 24.16]

*CI, confidence interval*.

First, the differences between the perception of selfies and photos (*H1a–H1f*) were considered. The results revealed a significant difference between selfies and photos with regard to perceived extraversion. Persons in selfies were rated as more extroverted than those in photos [*F*_(1, 289)_ = 15.90, *p* < 0.001, η^2^_*P*_ = 0.052], which supported *H1a*. There was also a significant difference for perceived openness [*F*_(1, 289)_ = 8.73, *p* = 0.003, η^2^_*P*_ = 0.029]. However, this turned out to contradict *H1b*, which assumed that persons in selfies would be evaluated as more open than persons in photos. With regard to *H1c*, no significant difference emerged between the perceived physical attractiveness of individuals in selfies and photos [*F*_(1, 289)_ = 0.93, *p* = 0.336, η^2^_*P*_ = 0.003]. However, a significant difference was detected for perceived social attractiveness between selfies and photos [*F*_(1, 289)_ = 7.60, *p* = 0.006, η^2^_*P*_ = 0.026], supporting *H1d*. Consequently, persons in selfies were rated as less socially attractive than persons in photos. In support of *H1e*, individuals in selfies were perceived as more narcissistic than in photos [*F*_(1, 289)_ = 27.06, *p* < 0.001, η^2^_*P*_ = 0.086]. Furthermore, a significant difference was found for the perception of trustworthiness between selfies and photos: Persons in selfies were rated as less trustworthy than persons in photos [*F*_(1, 289)_ = 19.67, *p* < 0.001, η^2^_*P*_ = 0.064]. Beyond the hypothesis, the above-mentioned factor “self-seeking” was included in the model. A significant difference in terms of type of picture can be seen, insofar as persons in selfies were rated as more “self-seeking” than persons in photos [*F*_(1, 289)_ = 28.82, *p* < 0.001, η^2^_*P*_ = 0.091].

Next, we focused on the question whether the gender of the pictured person affects the attribution of narcissism and trustworthiness depending on the portrayal in a selfie or photo (*H2a* and *H2b*). In general, we found a significant difference between the observation of females and males concerning perceived narcissism and perceived trustworthiness. Accordingly, males in pictures were rated as more narcissistic than females in pictures overall [*F*_(1, 289)_ = 4.55, *p* = 0.034, η^2^_*P*_ = 0.016]. Additionally, males were rated as less trustworthy than females [*F*_(1, 289)_ = 7.63, *p* = 0.006, η^2^_*P*_ = 0.026]. However, there were no significant effects regarding the interaction of gender and type of photo—either for perceived narcissism [*F*_(1, 289)_ = 0.05, *p* = 0.824, η^2^_*P*_ = 0.000] or for trustworthiness [*F*_(1, 289)_ = 0.16, *p* = 0.692, η^2^_*P*_ = 0.001]. Therefore, although males were indeed rated as more narcissistic and less trustworthy in selfies, the same was true for photos. Therefore, hypotheses *H2a* and *H2b* were not supported. Again, the additional measure “self-seeking” revealed a significant difference, as males in both photos and selfies were rated as more “self-seeking” than females [*F*_(1, 289)_ = 5.59, *p* = 0.019, η^2^_*P*_ = 0.019]. Additionally, there were significant differences regarding physical attractiveness [*F*_(1, 289)_ = 51.28, *p* < 0.001, η^2^_*P*_ = 0.151], social attractiveness [*F*_(1, 289)_ = 12.8, *p* < 0.001, η^2^_*P*_ = 0.042], and openness [*F*_(1, 289)_ = 5.03, *p* = 0.026, η^2^_*P*_ = 0.017]. Recipients perceived females in pictures as more physically attractive, more socially attractive, and more open than males in pictures.

To test hypotheses *H3a*–*H3d*, we examined the main effects of the condition “number of displayed persons” on perceived physical attractiveness as well as perceived social attractiveness. The MANOVA revealed no significant differences to support our assumptions. Profile owners who post group pictures were not evaluated as more physically attractive [*F*_(1, 289)_ = 0.28, *p* = 0.596, η^2^_*P*_ = 0.001), and nor as more socially attractive [*F*_(1, 289)_ = 1.53, *p* = 0.218, η^2^_*P*_ = 0.005], than isolated individuals in pictures. Also, the interactions between the number of shown persons and the type of picture were insignificant [*F*_(1, 289)_ = 1.46, *p* = 0.228, η^2^_*P*_ = 0.005 for perceived physical attractiveness and *F*_(1, 289)_ = 0.17, *p* = 0.685, η^2^_*P*_ = 0.001 for perceived social attractiveness]. Additionally, no significant result for the “self-seeking” factor was found [*F*_(1, 289)_ = 1.09, *p* = 0.299, η^2^_*P*_ = 0.004].

Although we did not expect to find specific interactions between all variables, a significant three-way interaction with regard to trustworthiness emerged [*F*_(1, 289)_ = 5.84, *p* = 0.016, η^2^_*P*_ = 0.02]. Among the single pictures, females were evaluated as more trustworthy when they showed photos, whereas both sexes were regarded as less trustworthy when showing selfies. Among the group pictures, selfies by male profile owners led to the lowest evaluations of trustworthiness.

### Mediation analysis

**Figure 2:**
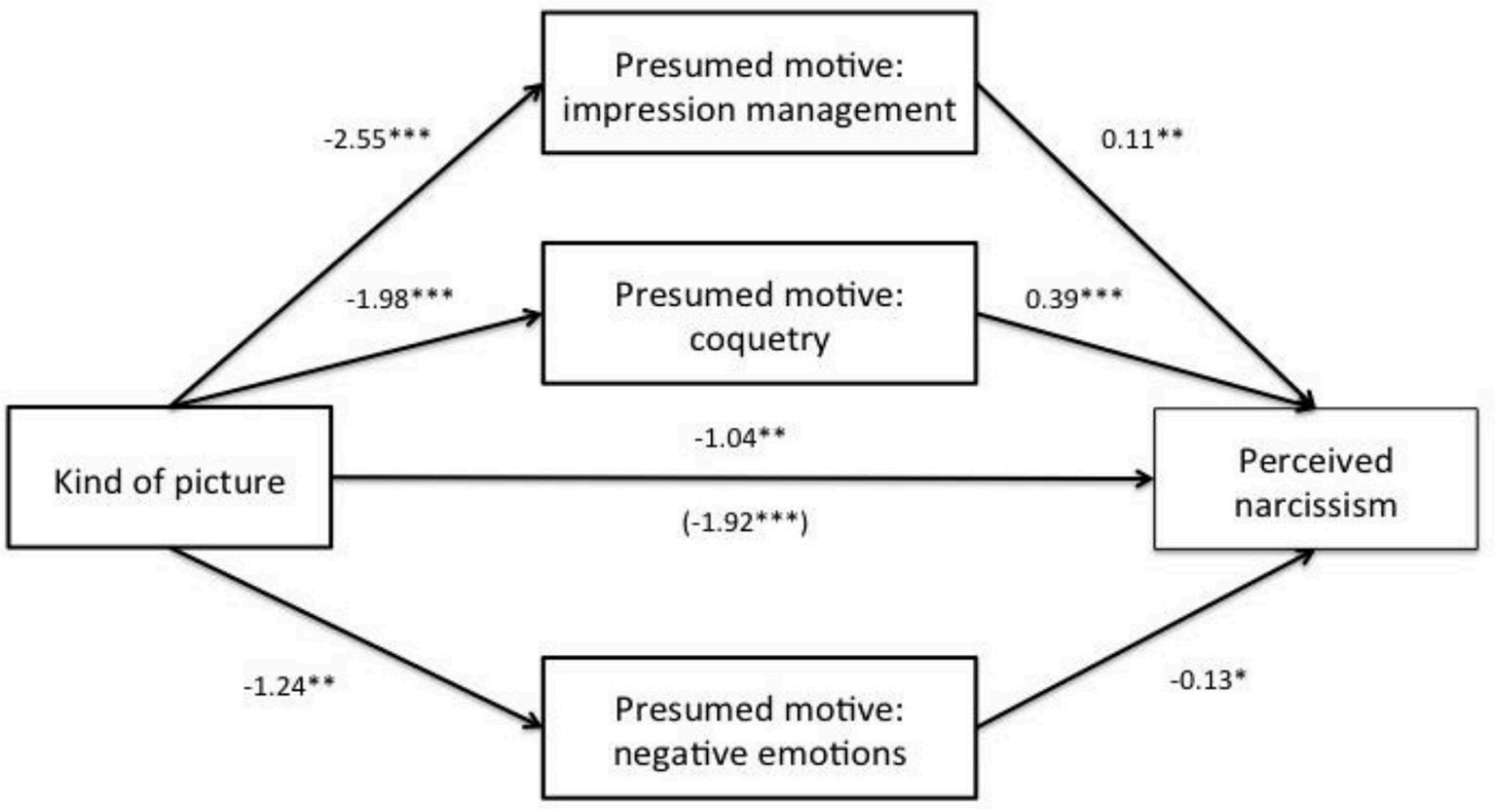
**Presumed motives for posting pictures as mediators of the effect of type of picture on perceived narcissism**. The value within parentheses represents the effect of the relation between the independent variables on the dependent variable before controlling for the mediator variables. Unstandardized coefficients are reported. ^*^p < 0.05. ^**^p < 0.01. ^***^p < 0.001.

The INDIRECT macro for SPSS (Preacher and Hayes, 2008) was supplementarily utilized to test the mediation hypothesis. We assumed that the impact of the independent variable type of picture on the dependent variable perceived narcissism would be mediated by the perceived motivations for posting pictures. The macro was used to calculate OLS regression analyses in order to examine whether possible indirect effects are still demonstrated by using bootstrapping. A significant indirect effect is given when the bootstrap confidence interval does not include zero, based on 5,000 bootstrap samples (with a percentile-based 95% CI; Preacher and Hayes, 2004). Following the recommendation by Darlington and Hayes (2016), we report unstandardized coefficients by using dichotomous independent variables. The mediation model is shown in Figure 2.

In the mediation model, all three motivational factors that were obtained by the EFA were included (impression management, negative emotions, coquetry). The independent variable type of picture predicted all mediators significantly. Likewise, the mediators were significantly related to the dependent variable perceived narcissism. The type of picture also predicted perceived narcissism significantly. A partial mediation effect was found for the relationship between type of picture and perceived narcissism. This effect occurred when controlling for the presumed motives as mediators, in that the impact of type of picture on perceived narcissism became smaller (*b* = −1.04, *p* = 0.003). Based on the bootstrap sample (5,000), the indirect effect was −0.88 (95% CI = [−1.34, −0.48]) for the overall model. In this respect, the presumed motive impression management showed an indirect effect of −0.27 (95% CI = [−0.52, −0.06]) and the presumed motive coquetry −0.78 (95% CI = [−1.21, −0.42]). The indirect effect of the presumed motive negative emotions was not significant 0.17 (95% CI = [−0.003, 0.40]). In sum, the results demonstrate that the presumed motive impression management and the presumed motive coquetry mediate the relationship between the independent variable type of picture and the dependent variable perceived narcissism.

## Discussion

The present study aimed to examine the potentially ambivalent reception of selfies in comparison to conventional photos. While there is an emerging body of research on taking selfies, this is one of the first studies to investigate the facet of perception and the question of how observers evaluate people shown in selfies. Our experiment employed mockups of Facebook profiles that varied in the type of picture (selfie vs. photo), sex of the profile owner, and the number of displayed persons.

The results reveal several notable differences between the reception of selfies and photos taken by other persons. In sum, people in selfies are perceived to be more extroverted, less open, less socially attractive, less trustworthy, and more narcissistic compared to the pictures that are photos taken by another person. In line with the Brunswik lens model (Brunswik, 1956), one could argue that the picture type is an important cue for the impression formation of a person's profile on Facebook and that posting selfies could thus be seen as a positive cue for extraversion and narcissism and as a negative cue for openness, social attractiveness and trustworthiness. The findings reveal that persons in selfies were perceived as more extroverted than those in photos. While it has been previously demonstrated that extraversion is indeed a predictor of selfie-posting behavior (Sorokowska et al., 2016), it is remarkable that observers seem to sense this. However, previous research already suggested that people use cues (in this case the number of friends) to infer extraversion of the profile owner (Hall and Pennington, 2013). Selfies, therefore, seem to be taken as a similar indicator of extraversion, which is also in line with general findings that show an actual relation between people's degree of extraversion and their posting behavior (Correa et al., 2010). With regard to openness to new experiences, we found the opposite effect of what we expected: Persons in photos taken by others were rated as more open than persons in selfies. As selfie-taking can be regarded as a comparatively new experience, it is remarkable that in this study, persons in regular photos were perceived as more open to new experiences. The fact that persons can control the way they appear in selfies to a greater extent than in regular photos might explain this lower level impression of openness: A person who is more willing to share pictures with uncertain outcomes may be seen as more open. Future research should therefore try to replicate this finding and address how it might be explained.

With regard to the question of whether the portrayal in selfies also yields a different evaluation than the portrayal in photos, we assessed perceived physical and social attractiveness, assumed narcissism, and assumed trustworthiness. While results regarding social attractiveness, narcissism and trustworthiness consistently, and in line with the hypotheses, show that people posting selfies are rated less favorably, there was no difference concerning physical attractiveness. This lack of difference is in contrast to findings of Re et al. (2016), who revealed that individuals in photos are rated as more (physically) attractive than individuals in selfies. Nevertheless, it is important to mention that in the study by Re et al. (2016), participants had to rate a wide range of individuals in selfies and photos taken by others. In our study, we presented each participant with only one person in the form of a Facebook profile owner. This might be a more accurate method, as it was shown that previously viewed faces affect the perception of attractiveness of other faces (Cogan et al., 2013; Pegors et al., 2015). Furthermore, Re et al. (2016) mixed the selfie and photo condition during the rating sessions, which might have caused an awareness of the research topic, and thus influenced the rating behavior. Another explanation may lie in the poses that were shown on the pictures in our study: In order to isolate the effect of the mere picture type, our models were instructed to show the same facial expression in the selfie and the photo. As a consequence, there were no selfie-typical gestures (e.g., perspectives, posing, duckfacing, hand gestures) and the selfies were more similar to the photos. Related to physical attractiveness, this would mean that the differences in perceived attractiveness might not be due to photo type but might result from selfie-specific poses.

Given this experimental control, by which we tried to isolate photo type from selfie-specific behavior, our results on perceived social attractiveness, narcissism and trustworthiness are all the more remarkable. The results show that observers are indeed suspicious when they sense that people are presenting themselves by means of a self-taken picture—even when the pose with which they present themselves is identical. This can be interpreted as in line with the warranting principle, as suggested by Walther et al. (2009). In our study, participants might have identified selfies as self-generated and photos as other-generated information. Following this line of reasoning, persons would distrust selfies more than photos taken by others because selfies seem to be easier to manipulate as they are generated by the profile owner her or himself.

The probably most important effect was observable on narcissism, as this dependent variable yielded the largest effect sizes. The results indicate that people might view individuals who post selfies as more narcissistic. The finding is in accordance with Re et al. (2016), who found the same difference in ratings of photos and selfies concerning narcissism but no difference in self-reported narcissism values. The latter authors suggested that selfies, with their self-promotional nature, transmit the impression that their producers are narcissistic.

With regard to narcissism, the mediation analysis revealed that the perceived motivations for picture posting partially account for finding that people posting a selfie rather than a photo are perceived as more narcissistic. In line with our theoretical considerations, the assumption that impression management is the motive for posting selfies leads to detrimental effects in the sense that increased narcissism is attributed. Furthermore, it is unsurprising that the presumed motive of coquetry contributed to explaining the relationship between picture type and perceived narcissism. Presumed emotional motives, however, do not seem to play a major role. Altogether, our findings show that motivational aspects not only play an important role regarding SNS usage (e.g., Krämer and Winter, 2008; Nadkarni and Hofmann, 2012; Tosun, 2012; Sung et al., 2016), but are also a factor that individuals take into account when forming an impression about other SNS users, and should therefore not be neglected in future studies. In this respect, external motivators like social expectations or peer pressure should also be taken into account.

Unlike the hypotheses on evaluation of selfies vs. photos, our assumptions on the interaction of gender and picture type were not supported. Although males in selfies were indeed rated as less trustworthy than females in selfies, this was also true for male and female profile owner in photos taken by others. Nevertheless, this main effect with regard to gender might fit the (production-related) finding that females post (Dhir et al., 2016) and take more selfies than males (Sorokowski et al., 2015, 2016; Sorokowska et al., 2016). Here, future research should try to replicate this finding that gender differences are more pronounced than potential interaction effects of gender and picture type.

Furthermore, the number of the displayed persons in selfies and photos did not lead to differences in the perceived physical and social attractiveness of the persons, which was contrary to our expectation. Persons in group photos and group selfies were not perceived as more physically attractive than single persons in photos and selfies—which is in contrast to results by Walker and Vul (2014), who showed that individual persons in group photos are viewed as more attractive than the same persons isolated in one photo. In addition, group photos and group selfies were not perceived as more socially attractive. These results contradict previous findings that the number of social cues in profile photos on Facebook is positively correlated with the perceived social attractiveness (Milyavskaya et al., 2010; Hong et al., 2012). One possible explanation for our finding might be that we asked the participants to concentrate on the evaluation of the profile owner. It is possible that this request caused participants to blank out the other persons in the group selfies and group photos. Moreover, the profile owner was pictured alone in the main profile photo in all conditions. The profile photo certainly has a special importance. This picture is usually the first impression and can be seen by anybody regardless of privacy settings (Hum et al., 2011). Profile pictures are thus the most important attempt to present oneself to the community (Ellison et al., 2006). In turn, this might have led SNS users (in our sample, 88.2% indicated having a Facebook account) to pay special attention to other people's profile photos. Since the assessment of attractiveness in our study occurred retrospectively and not while viewing the stimulus material, it is conceivable that this picture was the most present heuristic in order to evaluate the profile owners' attractiveness. Nevertheless, these findings remain surprising given the number of studies which have indicated a “cheerleader effect” (Walker and Vul, 2014) and should be explored further.

Considering the attention-grabbing nature of selfies (Souza et al., 2015) it can be speculated that the usage of feedback features (e.g., likes or comments) in case of selfies is not necessarily related to the individual perception and may therefore serve rather as a social strategy as opposed to a strategy for expressing honest evaluations (c.f. Lee et al., 2014, 2016). However, selfies are an aspect of the current pop culture (Barry et al., 2017) and our results do not preclude that they also might lead to positive outcomes. Future research should therefore explore the possibility of both positive and negative effects of selfies for those who post selfies. While in the case of our study participants evaluate selfies of strangers, it is conceivable that individuals evaluate selfies of familiar persons more positively. Nevertheless, selfie takers should be beware that selfies might not lead to desired attributions.

### Limitations and future research

As the present study is one of the first to investigate the perception of selfies, the presented findings should be treated with caution. Although we were able to corroborate previous results (Re et al., 2016) using a more controlled and systematic investigation, and found a consistent pattern of results regarding the detrimental effects of selfies, future research is required to replicate our findings. As the Brunswik lens model (Brunswik, 1956) would predict, selfies serve as a positive cue for the producer's extraversion and narcissism and as a negative cue for the producer's openness, social attractiveness and trustworthiness from the recipient's perspective. Future research should investigate how these cues interact with other cues which are positively correlated with social attractiveness and extraversion, such as number of friends (Tong et al., 2008; Hall and Pennington, 2013). To this aim, future research should concentrate on combining both perspectives—the recipient's and the producer's view—in a multi-method approach. Such an approach could additionally reveal how perceived and self-assessed personality traits might differ when comparing selfies and photos—similar to the investigation by Re et al. (2016).

Regarding the age distribution of the participants, more than 75% of participants were young adults between 15 and 29 years. Therefore, results cannot be generalized for the whole population. Future studies need to include more users older than 29 years. Still, the sample does not necessarily have to represent the whole population as the relevant user groups who will primarily get in touch with selfies on SNS are younger than the general population. Also, the results revealed only small to moderate effect sizes. It should also be noted that the sample contained more female participants than male participants, which might have skewed the results. Moreover, the time for which the participants viewed the profile needs to be considered. In this sample, a cut-off score of 5 s or less was set. It might be questioned whether 5 s are sufficient to build an impression of a person's profile. Most importantly, the data of the manipulation check suggest that participants did not perceive the profiles as predominantly composed of selfies vs. photos. Although the conditions did differ with regard to the remembered number of selfies/photos, participants believed that they remembered a substantial number of photos in the selfie condition, and conversely, a relatively high number of selfies in the photo condition. The main reason for this might be an artifact provoked by the corresponding items: Participants were asked in both conditions how many photos and how many selfies they had seen. Therefore, it is likely that they believed that both types of picture were presented to them. If these data are not merely an artifact but an indication that the number of selfies and photos was not consciously perceived by the participants, the obtained results would be all the more impressive.

In summary, our results reveal a counterintuitive pattern: Although selfies are a highly popular means of impression management, the findings suggest that they are less successful in achieving the goal of a positive impression than conventional photos. Therefore, when taking out their smartphones, SNS users who are striving for positive self-presentation and positive evaluations should be aware that their selfie might backfire—and they might be best advised to ask someone to take a photo of them.

## Ethics statement

All subjects gave written informed consent in accordance with the Declaration of Helsinki. The protocol was approved by the local institutional review board of the Department of Computer Science and Applied Cognitive Science.

## Author contributions

Conceptualization: NK. Formal analysis: MF, JK, YM, and MR. Investigation: MF, JK, YM, and MR. Methodology: SW, NK, MF, JK, YM, and MR. Resources: NK. Supervision: SW and NK. Writing—original draft: MF, JK, YM, and MR. Writing—review and editing: NK and SW.

### Conflict of interest statement

The authors declare that the research was conducted in the absence of any commercial or financial relationships that could be construed as a potential conflict of interest.
